# Can live-attenuated SARS-CoV-2 vaccine contribute to stopping the pandemic?

**DOI:** 10.1371/journal.ppat.1010821

**Published:** 2022-09-21

**Authors:** Patrick Chun Hean Tang, Wern Hann Ng, Nicholas J. C. King, Suresh Mahalingam

**Affiliations:** 1 Emerging Viruses, Inflammation and Therapeutics Group, Menzies Health Institute Queensland, Griffith University, Queensland, Australia; 2 The Discipline of Pathology and Bosch Institute, School of Medical Sciences, Sydney Medical School, The University of Sydney, Sydney, New South Wales, Australia; 3 Marie Bashir Institute for Infectious Diseases and Biosecurity, Sydney Medical School, The University of Sydney, Sydney, New South Wales, Australia; University of Colorado Denver, UNITED STATES

Coronavirus Disease 2019 (COVID-19) has plagued the world throughout 2020 to 2022, with over 580 million confirmed cases and 6.4 million deaths. Several vaccines have been approved for human use, including those from Pfizer-BioNTech, Moderna, Novavax, AstraZeneca, Johnson & Johnson, and Sinovac. The focus on rapid vaccine platforms such as mRNA, adenovirus vectors, DNA vectors, inactivated and subunit vaccines for COVID-19 [1] allowed the introduction of COVID-19 vaccines for human use in record time. These first-generation vaccines are major achievements, but improvements will be required to fully defeat this pandemic. In particular, new vaccines that deliver longer-lasting protection and provide broader immunity effective against current and future variants would be very valuable. Vaccines with a lengthy development pathway, such as the live-attenuated whole virus vaccines (LAVs) platform were largely overlooked in the race to develop a COVID-19 vaccine. A LAV is being developed by Codagenix in partnership with the Serum Institute of India and this single dose, intranasal vaccine has completed Phase I clinical trials, with promising safety and immunogenicity data [2]. There is a small number of other reported LAV that have shown utility in animal models of COVID-19 [3,4], such as a temperature-sensitive Severe Acute Respiratory Syndrome Coronavirus 2 (SARS-CoV-2) mutant that is able to replicate in the upper, but not the lower, respiratory tract [3]. Although development of LAV is time-consuming, they have several important advantages that make this approach attractive. In this article, we discuss the arguments in support of LAV as COVID-19 vaccines, together with some of the challenges facing their development and use.

## Successful track record against human viral diseases

The majority of vaccines licensed to control or prevent human viral diseases are live-attenuated viruses [5]. LAVs generally mimic natural infection, resulting in robust, functional immunity that appears to be identical to that following natural infection, sometimes after a single vaccination (e.g., herpes zoster vaccine, yellow fever vaccine). These vaccines have been used successfully for many decades, and their continued use attests to their safety. Examples include yellow fever vaccine, which has been available for over 80 years and provides lifelong immunity; oral polio vaccine, available since the 1960s, playing a crucial role in eradication of polio; and smallpox vaccine, which is based on vaccinia virus, a closely related LAV virus. Other important LAVs in routine human use include measles, mumps, rubella, chickenpox, and oral rotavirus. LAVs are generally unsuitable for those with compromised immunity, but there are already a number of COVID-19 vaccines in the market suitable for this group of individuals.

### LAV can induce both mucosal and systemic immunity similar to natural infection

Natural infection with SARS-CoV-2 induces broad T cell and humoral immune responses [6,7]. A number of epidemiological studies have shown that individuals previously infected with SARS-CoV-2 have an 80.5% to 100% decrease in their risk of a repeat infection [8]. Immunity induced by natural infection is of reasonably long duration. The presence of protective SARS-CoV-2-specific memory B and T (CD4^+^ and CD8^+^) cells were observed 3 months after mildly symptomatic COVID-19 [9], while specific memory T cells were present after mild or severe COVID-19 [10]. Dan and colleagues followed antigen-specific antibodies, memory B cells, CD4^+^ T cells, and CD8^+^ T cells up to 8 months after recovery from COVID-19 [11], but a more recent study has shown sustained T and B cell responses over a year after infection [12]. In this latter study, despite reducing RBD-specific IgG and neutralising antibody titres between 3 and 6 months post infection, specific memory B cells plateaued over the remaining 6 months, with functional antibodies giving a predicted 45% to 76% protection against 4 SARS-CoV-2 strains studied. In general, disease severity enhanced these responses [12], as has previously been observed [13,14]. The antibody response following SARS-CoV-2 infection also appears to be largely effective against most emerging variants [15]. Very recent research suggests that immunity due to previous natural infection with SARS-CoV-2 is superior to that induced by BNT162b2 vaccination. In a retrospective study of a cohort of almost 25,000 patients, fully vaccinated (2 doses of Pfizer BNT162b2) individuals had a 13.06-fold increased risk of breakthrough infection, compared to those who had been naturally infected. Even accounting for waning immunity after natural infection, the risk in vaccinees was still 5.96-fold more than in naturally infected individuals [16]. Thus, natural immunity was stronger and longer-lasting than in BNT162b2 vaccinees and provided superior protection against infection, symptomatic disease, and hospitalisation caused by the SARS-CoV-2 delta variant. Furthermore, survivors of SARS-CoV-1 boosted with BNT162b2 vaccine, in addition to strong neutralisation of the range of medically important SARS-CoV-2 variants, showed highly elevated cross-clade neutralising Ab responses to several Sarbecoviruses after a single boost, suggesting that live virus infection likely primes for an accelerated cross-reactive response more capable of neutralising emerging variants than previously suspected [17]. Nevertheless, it is important to emphasize that in general, neither natural infection or immunisation prevents re-infection per se, but the accelerated adaptive memory responses significantly reduce disease and symptoms, if not entirely abrogating them. As a result, transmission, if not prevented, is also considerably temporally reduced [18].

SARS-CoV-2 is a respiratory pathogen that infects mucosal surfaces. Replication at the mucosal surfaces leads to development of strong mucosal immunity, which is particularly effective in preventing subsequent infections, although this wanes quickly compared to the lifelong immunity induced by more systemic viral infections, despite their similar use of the respiratory tract as the portal of infection and transmission in many cases [19]. Current COVID-19 vaccines do not induce strong mucosal immunity and the speed of virus mutation has complicated this still further. This is not surprising as they are administered by intramuscular injection far from the site of natural infection. While the current generation of COVID-19 vaccines induce strong immunity, emerging data suggest immunity starts to wane relatively soon after vaccination. For example, recent data have shown that total antibody levels can start to decrease as early as 6 weeks after completing immunisation with BNT162b2 and ChAdOx1 nCoV-19, with an expected reduction >50% over 10 weeks or within 2 to 3 months [21,22]. The inability of current vaccines to induce strong mucosal immunity, together with the relatively rapid waning of vaccine-induced immunity, is likely to contribute to breakthrough infections. Vaccinated individuals who experience breakthrough infections generally clear their infections faster and have milder disease than unvaccinated individuals. However, breakthrough infections are often accompanied by high viral shedding, comparable in many cases to the viral loads reported in unvaccinated individuals [23]. This is important since it suggests that vaccinated individuals with breakthrough infections are likely to transmit SARS-CoV-2 to others.

**Fig 1:**
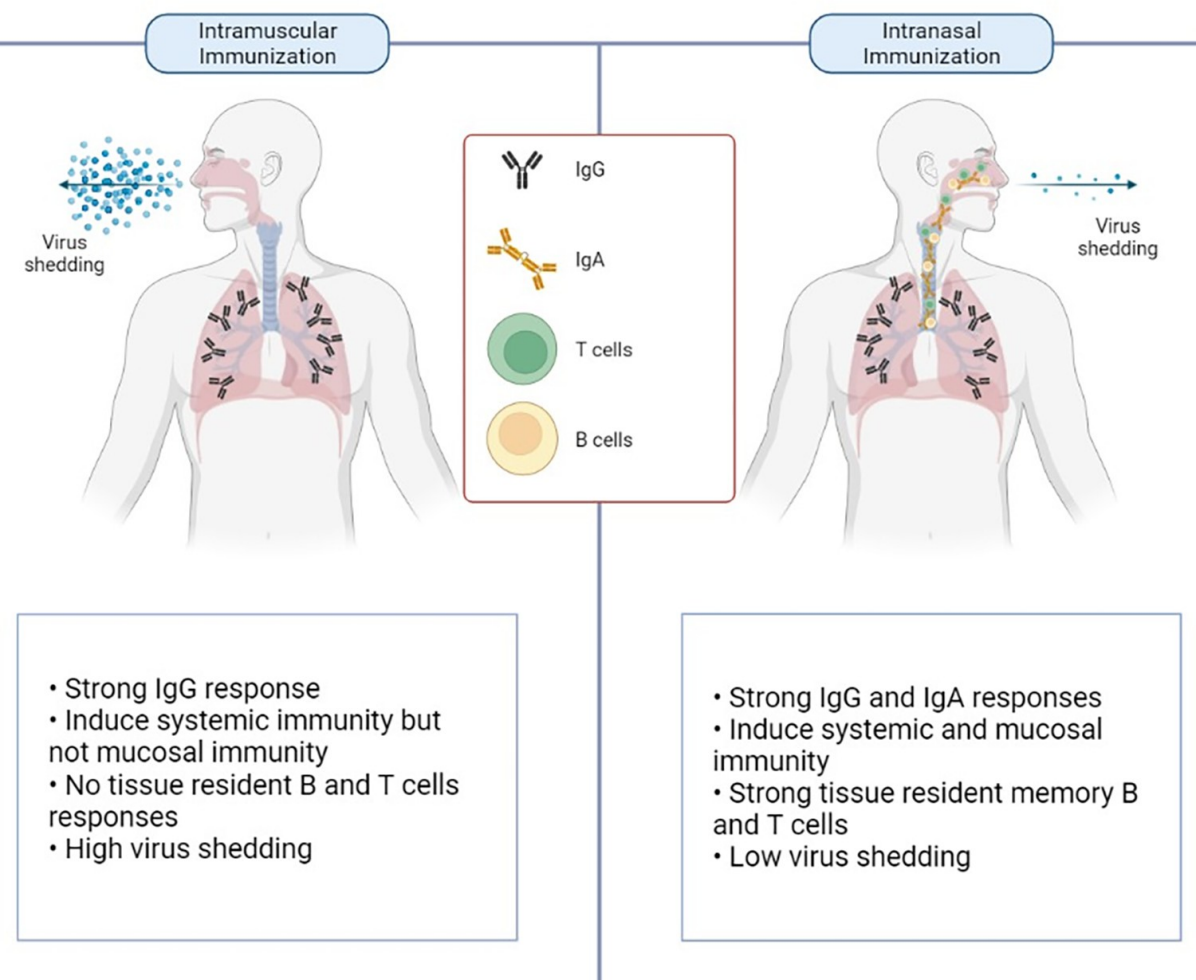
Key differences in immunity following intramuscular and intranasal immunization. This figure is modified from “Scent of a vaccine” in Lund and Randall, 2021 [20].

Intranasal immunisation is expected to induce both localised and systemic immunity, including IgA and IgG responses and resident memory B and T cells (Fig 1). The ability to deliver vaccines intranasally may provide a major advantage over other routes of immunisation due to enhanced mucosal immunity in the upper airways providing a strong first line of defence to infection. Intranasal vaccine delivery is potentially simpler, faster, and cheaper than alternative routes of immunisation, making this approach very desirable in the challenge of providing global vaccine coverage in the fastest possible time. In addition to the intranasal route of immunisation, LAV can also be administered subcutaneously. While this route alone is unlikely to induce such strong mucosal immunity, it is very effective for induction of systemic immunity and would provide a broad and comprehensive boost after primary vaccination at either site.

### LAV induces immunity against a range of SARS-CoV-2 proteins

Natural infection with SARS-CoV-2 results in strong immunity to a wide range of virus proteins in addition to spike, including the nucleocapsid protein, nonstructural proteins, and those encoded for by ORF1 [6,24,25]. The response is highly polyclonal, with antibodies against 22 distinct proteins/epitopes in the SARS-CoV-2 virion [26]. In addition to antibodies, SARS-CoV-2 infection induces a strong T cell response. Since viral proteins are present in the cytosol of infected cells, the T cell response includes CD8^+^ T cells, which have the potential to directly kill infected cells. Several studies have found CD4^+^ and CD8^+^ T cell memory responses to most SARS-CoV-2 proteins except for envelope protein, in individuals who had recovered from SARS-CoV-2 infection [11,27,28]. In contrast, vaccines that direct the immune response only to the spike protein induce a narrower response than vaccines that induce immunity to the live virus. Thus, emerging spike variants may more readily escape immunity induced by these vaccines, as they do not include the additional viral epitopes presented to the immune response in a natural infection, i.e., epitopes that would likely be recognised on challenge by a novel spike variant to enable the accelerated arrest of infection by memory responses. This potentially allows the perpetuation of transmission during breakthrough infections, as well as the development and establishment in turn of new SARS-CoV-2 variants, thereby maintaining a circulating quorum of SARS-CoV-2 in the community. This important advantage of LAVs is somewhat nuanced because not all SARS-CoV-2 proteins induce a neutralising antibody response; nonetheless, an antigenically broad immune response is likely to provide superior protection against emerging variants. To keep up with emerging variants, current vaccines targeting the S protein may have to be modified repeatedly, a challenging process on a global scale. Continued emergence of new variants is likely to require ongoing tests of each new “tailored” vaccine. By generating B and T cell epitopes from the full range of virus proteins, LAVs provide a broader immune response to SARS-CoV-2, the efficacy of which is less likely to be affected by alterations in single regions of the virus. Although not all virus proteins may induce strong neutralising antibody responses, other arms of the immune response also provide meaningful protection. In particular, the ability of LAVs to induce strong CD8^+^ T cell responses has the potential to provide strong protection from SARS-CoV-2 infection. Antigenically broad immunity induced by LAVs may allow this vaccine type to provide cross-protection against antigenically more distant strains of the targeted pathogen. Recently, it has been shown that human tonsils from COVID-19-naïve individuals contain CD8^+^ tissue-resident memory T cells (T_RM_) cross-reactive for SARS-CoV-2 [29]. Intranasal immunisation with LAVs should induce a broad range of SARS-CoV-2-specific T_RM_ that would be strongly protective as well as likely cross-reactive to new variants [30,31]. Infection at the mucosal site is also likely to enable accelerated local innate cellular responses that are protective early in rechallenge infection [32]. We are mindful that development of antibodies against conserved regions of SARS-CoV-2 may not necessarily provide protection against variants if those conserved regions are not directly accessible to antibodies or exposed early in the process of host cell infection. However, in addition to antibody responses, as long as these conserved regions are presented to the immune response, they could deliver cell surface epitopes for recognition by effector and memory T cells, expediting virus eradication and potentially forestalling onset of the damaging hyperinflammatory myeloid phase.

### Modern molecular approaches to prevent reversion to virulence

The traditional approach to develop successful LAVs has been serial passage of virus in hosts that do not normally support infection or disease. This empirical approach has been successful, although reversion to virulence may occur and must be carefully monitored. Efforts to develop a live-attenuated dengue serotype 4 vaccine were plagued by phenotype instability and reversion to wild-type pathogenicity when viruses were grown in fetal rhesus monkey kidney cells [33]. In addition, reversion of dengue serotype 3 was responsible for ending the Aventis/Mahidol vaccine initiative [34]. Instability and reversion to pathogenicity of polio vaccines has been a problem requiring sustained management, although oral polio vaccines are still in use today. Modern molecular approaches to vaccine attenuation such as codon deoptimization are replacing blind serial passage and have been enabled through the development of reverse genetics systems that allow the introduction of large numbers of precisely targeted attenuating mutations in genes coding for nonstructural proteins that regulate virulence or replication [35]. By introducing large numbers of mutations, the probability of reversion to the virulence of the original wild-type virus is greatly reduced. In addition to their superior safety, such approaches may also be faster and more cost-effective than traditional LAV methodologies. Nevertheless, it is essential to undertake extensive screening to fully evaluate attenuation and genetic stability of the attenuated genotype.

### Scenarios in which LAV would be beneficial

While current generation COVID-19 vaccines appear to be highly effective in preventing severe disease, we believe vaccines that are durably and broadly protective will be required to reduce transmission and protect against emerging variants. Many LAVs have been shown to meet these requirements and efforts to derive live-attenuated SARS-CoV-2 vaccines should have a high priority. There are several key scenarios in which the advantages of LAV will be particularly important. First, it is becoming increasingly apparent that inactivated COVID-19 vaccines, which are the most widely used vaccine type globally (e.g., Coronavac and Sinopharm vaccines), provide very limited protection against, e.g., the omicron variant. LAV boosters may be particularly effective in recipients of inactivated vaccines by inducing robust, long-lived immunity that is likely to provide additional protection against omicron. Second, LAVs are likely to induce broad immunity in a similar manner to that seen in natural SARS-CoV-2 infection. The immune response in vaccinated individuals who have also been infected with SARS-CoV-2 is particularly robust and effective [36]. Thus, it is very likely that the immune response in individuals vaccinated with a LAV and boosted, either with LAV or spike-specific vaccines, will strengthen and broaden this response still further, as observed with BNT162b2 vaccine boost after SARS-CoV-1 infection, concomitantly providing protection against future emerging variants. Thirdly, LAVs given intranasally would have the further advantage of priming early protective innate cellular responses, as well as inducing localised adaptive memory, both rapidly activated by subsequent upper respiratory tract virus infection. Multilayered innate and adaptive antiviral responses generated by a combination of temporally staged vaccinations that include a LAV, could be particularly effective against emerging variants likely to be most frequently and repeatedly encountered by frontline workers, substantially mitigating the risk of transmission into the community.
